# Social Support and 10-Year Mortality Following Acute Myocardial Infarction

**DOI:** 10.3390/jcdd12040147

**Published:** 2025-04-10

**Authors:** Batya Betesh-Abay, Arthur Shiyovich, Ygal Plakht

**Affiliations:** 1 Department of Nursing, Faculty of Health Sciences, Ben-Gurion University of the Negev, Be’er Sheva 8410501, Israel; tobibetesh@gmail.com; 2 Department of Cardiology, Rabin Medical Center, Petah Tikva 4941492, Israel; arthur.shiyovich@gmail.com; 3 Sackler Faculty of Medicine, Tel Aviv University, Tel Aviv 6997801, Israel; 4 Division of Cardiovascular Medicine, Department of Medicine, Brigham and Women’s Hospital, Harvard Medical School, Boston, MA 02115, USA; 5 Department of Emergency Medicine, Soroka University Medical Center, Be’er Sheva 8410101, Israel

**Keywords:** myocardial infarction, mortality, social support, outcomes

## Abstract

This study investigates social support type and long-term mortality following AMI. Demographic and clinical data were collected retrospectively from a tertiary hospital for all patients with AMI (2011–2017). Study groups based on support type were defined: (1) employed partner (served as the reference group); (2) unemployed partner; (3) no partner, family support; (4) institutional or benefit-dependent; and (5) non-kin support (caregiver). Ten-year all-cause mortality risk was assessed and compared between the groups. We identified 2652 AMI patients with recorded support type: mean age 67.6 (SD = 14) years, 66% male; 40% had no partner, followed by those with an unemployed partner (31%). Over the follow-up of (median) 7.6 years, 1471 patients died; significantly higher mortality rates were observed in patients without family support (67.9%) or receiving non-kin support (94.9%). Those with non-kin support were at the highest mortality risk, AdjHR = 2.20, 95% CI: 1.67–2.91, *p* < 0.001, as compared with the reference group. Subgroup analyses found women below age 75 years, Arab women, and those with higher functional status to be most vulnerable to mortality in the absence of family support. Lack of family support was associated with increased long-term mortality among AMI patients. Assessment of support status among AMI patients is integral for secondary prevention.

## 1. Introduction

Cardiovascular disease accounts for a tremendous global burden, leading to strenuous expenses, morbidity, mortality, and emotional and physical hardship [1,2,3]. Acute myocardial infarction (AMI), in particular, is among the most prevalent form of coronary artery disease [4]. Reduction of AMI-related death has been obtained by identification and modulation of established risk factors including smoking, dyslipidemia, diabetes mellitus, and hypertension [5,6]. Nevertheless, the development and progression of cardiovascular disease may be significantly influenced by sociological, psychological, and environmental factors.

The notion of social and emotional determinants being linked with physical health and mortality has attracted more attention in recent decades [7,8,9]. Social support denotes the emotional, functional, and informational resources provided by others as well as the quality of those resources [10,11]. Social support is a well-recognized determinant of health outcomes, with various assessment tools available. The Oslo-3 Social Support Scale (Oslo-3) is a widely used measure that evaluates perceived social support through three key questions, providing a concise yet reliable assessment of support levels [12]. Similarly, the Medical Outcomes Study (MOS) Social Support Survey is a comprehensive instrument that quantifies different dimensions of social support, including emotional, informational, tangible, and affectionate support, making it a valuable tool for understanding the broader implications of social networks on health [13].

Research examining elements of social support and their direct interconnection with cardiovascular health generally indicate that the absence of stable social support negatively impacts cardiovascular-related outcomes [14,15,16,17]. However, some works did not find this association [18]. Further, exactly which support type may be ideal remains unestablished; research examining familial support versus non-kin support show inconsistencies, with some reporting familial support to be favorable for survivability [19], and others finding non-kin support to be prognostically preferred [20,21,22], likewise with married [23,24,25,26,27] versus non-married individuals [28,29,30].

This study aims to examine social support types and their association with mortality in a ten-year time period following an AMI event.

## 2. Materials and Methods

### 2.1. Population and Setting

This retrospective study included adult (≥18-year-old) patients hospitalized at Soroka University Medical Center (SUMC) between 1 December 2011 and 31 October 2017 with a diagnosis of AMI. SUMC is a tertiary teaching hospital of ~1200 beds located in Beer-Sheva, Israel, predominantly serving the Southern District of Israel. Over 500,000 residents live in southern Israel, and approximately 35% of them are Muslim Arabs (Bedouins). Despite the geographic proximity, these ethnic groups greatly differ in their lifestyle, demographic growth, morbidity, and health-related outcomes [31,32,33].

This study is part of the Soroka Acute Myocardial Infarction (SAMI) project [34,35,36,37]. For patients with multiple hospitalizations during this period, the first admission was considered. Exclusion criteria were non-citizens of Israel, in-hospital mortality, and absence of documented social support information in electronic medical records. This study received institutional ethical approval, waiving the need for patient consent due to its retrospective nature.

### 2.2. Study Groups

Patients were grouped based on ‘support type’ as recorded in a single-choice dropdown field in the electronic medical charting system upon nursing admission assessment. Numeric identifiers were assigned to each support type and categorized as follows: *Group 1 (served as the reference group)*: employed partner; *Group 2*: unemployed/supported partner; *Group 3*: no partner, family support; *Group 4*: institutional or benefit-dependent (receives benefits or lives in a licensed care home/assisted living facility); and *Group 5*: non-kin support (home caregiver, senior social club, or nursing home).

### 2.3. Follow-Up and Outcome

The follow-up period was up to 10 years post-discharge or until 31 July 2023. The primary outcome was all-cause mortality during the follow-up period.

### 2.4. Data Collection and Definitions

Demographic and clinical data were extracted from electronic medical records. Classification of Diseases, Ninth Revision, Clinical Modification (ICD-9-CM) [38] was used to identify comorbidities as recorded by the attending medical staff during patient hospitalization, in accordance with predetermined criteria as detailed hereafter. Personal mortality data were sourced from the Israeli Ministry of the Interior Population Registry.

A diagnosis of AMI was established in alignment with the Universal Definition of Myocardial Infarction [39] as relevant at the time of data collection, defined as a combination of ischemic signs and/or symptoms along with a sudden increase and subsequent decrease in levels of cardiac biomarkers, indicative of acute myocardial injury. In addition to ICD-9-CM criteria, diabetes mellitus was defined as hemoglobin A1C (HbA1C) of ≥6.5% and dyslipidemia as low-density lipoprotein (LDL) levels ≥100 mg/dL at any timepoint throughout the 12-month period from six months prior to hospitalization. Obstructive coronary artery disease referred to detection of a ≥70% vessel stenosis confirmed by angiography. Severe left ventricular dysfunction was determined as a left ventricular ejection fraction of <30% on the first echocardiogram of hospitalization; pulmonary arterial systolic pressure of ≥37 mmHg on the same exam indicated pulmonary hypertension.

Additionally, Norton Scale (NS) scores [40] were obtained from patient medical charts, as assessed by nursing staff upon admission and reassessed as needed throughout hospitalization. If multiple assessments were conducted, the most recent score was utilized. The NS score (pressure ulcer risk) has been demonstrated [41,42] to be a prognostic indicator used to measure overall physical and cognitive function. The NS assesses five domains, physical condition, mental condition, activity, mobility, and incontinence, each scored on a Likert scale of one to four. Domain scores are summed, yielding a total that may range from 5 to 20 points; scores below 16 indicate increased pressure ulcer risk.

### 2.5. Statistical Analysis

Demographic and clinical patient data were evaluated across the support groups. Continuous variables are presented as mean and standard deviation (SD) or median and interquartile range (IQR). Comparisons were made using Analysis of Variance (ANOVA). Categorical variables are expressed as numbers (percentages), and their comparisons were conducted using either the Chi-square test or Chi-square test for linear trend.

Kaplan–Meier analyses present the time-to-event data and were compared using the Log-rank test. The relationship between support status and the outcome in the univariable and the multivariable levels was determined by Cox Proportional Hazards regressions. The results of the models are presented as hazard ratios (HR) and adjusted HRs (AdjHR) with 95% confidence intervals (CIs). Variables with a *p*-value of <0.1 in the univariable analysis were included in the multivariable model. In addition, we performed subgroup analyses, which estimated the relationships between the type of social support and the investigated outcome separately by sex, nationality, age, and functional status.

A two-sided *p*-value of <0.05 was considered statistically significant. Statistical analyses were performed using Statistical Package for the Social Sciences (SPSS) version 29 (IBM Corporation, Armonk, NY, USA).

## 3. Results

### 3.1. Study Population and Groups

During the data collection period, hospitalizations of 7375 patients were documented. The final cohort included 2652 patients (Supplementary Figure S1). The mean age of the study population was 67.6 (SD = 14) years, most (66%) were men, and about 20% were Arab minorities. The main risk factors included diabetes mellitus, dyslipidemia, smoking, and anemia. Non-ST elevation myocardial infarction (NSTEMI) was the most common presentation, and more than 70% of the patients received invasive treatment. About 22% of the study cohort had impaired functional status (NS < 16) (Supplementary Table S1).

The distribution of patients across support groups revealed that the largest study group was Group 3 (no partner), comprising 40.0% of the study population, followed by Group 2 (unemployed partner) at 31.0%. Group 1 (employed partner) made up 17.3%. The least represented groups were Group 5 (receives non-kin support) and Group 4 (benefit-dependent), constituting 5.9% and 5.8%, respectively.

### 3.2. Baseline Characteristics

Demographic and clinical parameters varied across the study groups (Table 1). Age differed significantly, with *Group 5* being of the oldest mean age. Significant differences were also found in sex distribution, with the highest proportion of males in *Group 1* (80.8%). Prevalence of cardiovascular risk factors, comorbidities, functional status, and AMI characteristics differed across groups.

### 3.3. Follow-Up and Outcome

**Figure 1:**
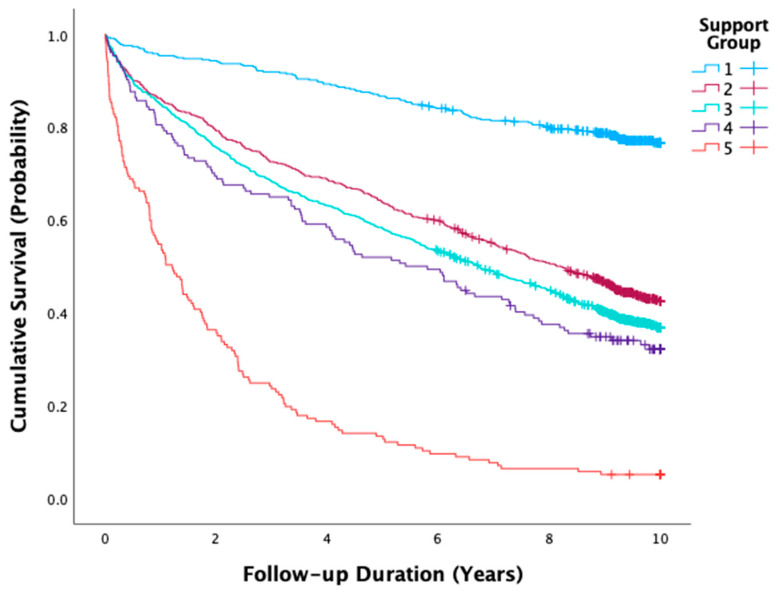
Kaplan–Meier estimates of survival following AMI event according to study group (Log rank test for all comparisons, *p* < 0.001). Support Groups: *Group 1*: employed partner. *Group 2*: unemployed partner. *Group 3*: no partner, family support. *Group 4*: institutional or benefit-dependent (receives benefits or lives in a licensed care home/assisted living facility). *Group 5*: non-kin support (home caregiver, senior-social club, or nursing home).

The follow-up period extended from three to 3652 days, with a median of 2789 days (~7.6 years) (IQR 853–3558 days). Over the course of the follow-up, 1471 patients died, yielding a mortality rate of 55.5%, and a cumulative mortality of 0.567. Table 2 displays mortality indices for the study population; survival curves for the study groups are illustrated in Figure 1. Statistically significant differences in mortality between the study groups were found. The highest mortality rate was in *Group 5* (94.9%), followed by *Group 4* (66.9%).

The univariate analysis, considering support type, revealed significant associations with all-cause mortality. Notably, all study groups displayed an increased mortality risk, compared to *Group 1*. *Group 2* HR 3.18 (95% CI: 2.57–3.94), *Group 3* HR 3.75 (95% CI: 3.05–4.61), *Group 4* HR 4.38 (95% CI: 3.33–5.75) and *Group 5* HR 13.49 (95% CI: 10.47–17.39), (*p* < 0.001 for all comparisons).

### 3.4. Multivariable Analysis

Table 3 presents the results of the multivariable analysis. A significant association between the type of social support and the risk for long-term all-cause mortality was found. As compared with the reference group, *Group 5* continued to demonstrate the highest mortality risk of all study groups: AdjHR 2.20 (95% CI: 1.67–2.91), *p* < 0.001. Additionally, several other variables showed significant associations with increased risk for mortality, including progressive age, impaired functional status, comorbidities (such as renal diseases, peripheral vascular disease, chronic obstructive pulmonary disease, malignancy, and alcohol/drug addiction), as well as a non-invasive treatment for AMI.

### 3.5. Subgroup Analyses

The distribution of support types varied significantly across the subgroups, as presented in Supplementary Table S2. Younger patients, men, and Jews were more likely to receive support from family members, while older patients, women, and Arabs were more inclined to receive non-kin support or have no partner. Better functional status, as indicated by higher NS scores, was associated with a greater percentage of family support.

The results of subgroup analyses are presented in Supplementary Table S3. Disparities in the strength of the relationship between type of social support and the risk of the outcome were observed between Jews and Arabs, with the highest mortality risk seen in Arabs of *Group 4* with AdjHR of 3.70 vs. AdjHR of 1.55 for this group in Jews. Among patients with NS scores equal to and above 16, increased mortality risk was found in all groups compared to the reference group, in contrast to those with lower NS scores, where no statistically significant relationships were found. Additional subgroup analyses demonstrated the highest risk for mortality in *Group 5* was among women below 75 years (AdjHR = 5.29) and Arab women (AdjHR = 7.62).

## 4. Discussion

The present study investigated the relationship between social support type and the risk of 10-year all-cause mortality following AMI. The major findings of the study were as follows: (1) 40% of included patients did not have support from a partner; (2) patients who were benefit-dependent or receiving support by non-family members exhibited significantly increased long-term mortality; (3) the association between social support type and mortality remained robust after adjusting for potential confounding variables, indicating an independent association; and (4) young women (below age 75 years), Arab women, and those with higher functional status were most vulnerable to mortality in the absence of family support.

This study contributes to the body of literature finding a direct interconnection between social support type and cardiovascular disease outcomes [14,15,16,17]. Among our cohort, we found that the distribution of patients across different support groups revealed a prominent prevalence of individuals with no partner (Groups 3–5), making up 40% of the study population. This finding emphasizes the notable portion of patients who are discharged after an AMI event and are lacking support from a domestic partner. Dhindsa et al. [43] comprehensively reviewed marital status and outcomes in patients with cardiovascular disease, elucidating that the unmarried have an increased incidence of adverse cardiovascular events when compared to their married counterparts. It has been suggested that these (unmarried) individuals are more likely to sustain depressive symptoms, exhibit low medication adherence, as well as face socioeconomic challenges that may negatively affect their health outcomes [44,45]. Furthermore, reports show that married individuals, or those who live with a partner, are less likely to smoke and more likely to engage in physical activity [28].

We designated ‘employed partner’ to be the reference category. This decision was based on findings deduced from literature [46] as well as the presumption that in such a case, both the patient and the partner likely function independently, and thus, this setup may be optimal for patients following AMI. Our results supported this hypothesis and further found that having an unemployed or dependent partner was associated with an increased mortality risk of 1.5-fold, connoting that perhaps the partner is not working for reasons pertaining to the health/physical function of one of the household members. In this regard, we added the NS score into our analyses; we intended to investigate if the absence of support from a partner or family members may be prognostically similar to impairment in physical/cognitive function. We indeed found that within our study model, those with lower NS scores demonstrated an increased mortality risk. This finding aligns with the literature in which lower NS scores, as well as functional impairment, serve as independent predictors of mortality [47]. However, our subgroup analysis revealed that among individuals with higher functional status (as indicated by higher NS scores), the relationship between support type and mortality was stronger compared to those with lower NS scores. This counterintuitive result might be explained by the hypothesis that functionally capable individuals require support that goes beyond instrumental assistance. We speculate that this additional support, which may be emotional or psychological, may be provided by family members.

We acknowledge the importance of pharmacologic treatment data, particularly dual antiplatelet therapy (DAPT), consisting of aspirin and a P2Y12 inhibitor, as a cornerstone of secondary prevention in AMI patients. These data are not available in our analysis but reasonably played a role in patient outcomes. Călburean et al. [48] showed that adjunctive therapies, such as eptifibatide and manual thrombus aspiration, did not improve long-term survival after STEMI in patients undergoing primary PCI. This underscores the importance of optimizing standard pharmacologic therapy, potentially including the appropriate use of DAPT, to enhance outcomes in AMI patients.

Differences in revascularization strategies also appear to play a role in patient outcomes. The lower PCI rates observed in certain subgroups, particularly in Group 5, likely reflect differences in AMI presentation and patient characteristics. Notably, in Group 5, only 8.9% of patients had STEMI, and of those, 64.3% underwent PCI, compared to 98.5% in Group 1. This figure may derive from functional impairment and lack of family or social support on treatment decisions, suggesting that these vulnerable patients may receive suboptimal care, including significantly lower rates of PCI.

We examined the findings of this study in the context of age, given that baseline characteristics of the study groups showed that those with an employed partner were of the youngest mean age and those receiving non-family support were of an older average age. To account for this potential bias, adjustment for age was applied, and subgroup analysis was performed. We found that significant differences were still observed in mortality HRs of those without family support, exhibiting a substantially increased independent mortality risk compared to the reference group, even among those below 75 years. Our results align with the literature. Bucholz et al. [16] examined social support and outcomes in young patients (aged ≤ 55 years) after AMI. Although they did not investigate mortality in their multivariable models, their findings revealed low social support to be associated with lower mental functioning, lower quality of life, and more depressive symptoms at 12 months post-AMI. Green et al. [49] investigated the usefulness of social support in older adults only (aged 75 years and older) following AMI hospitalization. Their findings showed low emotional support to correlate with mortality (odds ratio of ~1.5) among this age group.

In comparing our findings to other registries, a large study examining outcomes among Medicare beneficiaries found that despite overall improvements in 10-year AMI survival, significant disparities persisted among demographic subgroups; men exhibited higher long-term mortality than women, and Black patients had worse outcomes compared to White patients. Similarly, our study highlights the association between social and demographic factors on survival, reinforcing the need for targeted interventions to address disparities in long-term AMI outcomes [50].

Ethnicity has been associated with differences in cardiovascular outcomes across various populations. In our study, we observed variations in the association between social support and mortality among Arab patients, suggesting the relevance of ethnicity in post-AMI survival patterns. Similarly, Călburean et al. reported that the ethnic minority populations may experience disproportionately higher long-term mortality following ischemic heart disease, as demonstrated in a prospective PCI registry from Eastern Europe. The findings indicated significantly higher all-cause and cardiovascular mortality rates among under-resourced ethnic groups [51].

We found that the least favorable prognosis was given when there was no support from family and instead support from non-family members such as a caregiver or nursing home. These findings were consistent with some literature [19]. However, other works examining familial versus non-familial support and survival found non-kin support to be favorable over family support [20,21,22]. A possible explanation for this inconsistency may relate to cultural features of the local geographic population, in which predominant social support may be provided by extended familial networks living in close proximity [52,53], a lifestyle that may resemble elements of traditional living.

In the current study, we categorized the study groups based on the domains provided by the electronic charting system, as assessed by the nursing staff at intake upon patient admission. Although we perceived *Group 1* (employed partner) to be the reference category, all other support groups were designated in a nominal manner and are not intended to be assigned ordinally, representational of a hierarchy. Nevertheless, it may be reasonable to perceive *Group 1* as the optimal support type, and every succeeding group as less ideal than the one prior. In this context, multivariable analysis may have demonstrated a supposed ‘dose–response’ relationship, where decreasing levels of support are associated with a higher risk of mortality, even after adjustment for the investigated confounders (such as age, comorbidities, and functional status). This finding is conceptually consistent with previous reports [17,46]. However, given that the groups were assigned by the available support domains, as stated, we found that our study groups did not exactly correlate with those examined in other works of research. For example, several studies examined marital status [23,25,26,27,28], living arrangements [54,55,56], or employment/socioeconomic status [46,55] based on their defined criteria and group delegation, as available. Nevertheless, parallels may be drawn, finding that less support, of any type, leads to worsened prognosis for patients following AMI.

Our study contributes to the existing literature by providing insights into the interplay between support networks and mortality pertaining to cardiovascular disease. Investigation of these parameters is markedly different than examination of ‘classic’ biological/clinical markers which may be researched by defined universal cutoffs and clear quantifiable variables [1,6]. The findings of the present work and the relevant literature [17,46,55] show the importance of clinical consideration in regard to patients’ living arrangements, income source, and support status. Nevertheless, we found that only 38% of AMI patients in our cohort had documentation of this information. Health institutions and clinicians should consider the importance of intaking further information regarding patient social support, similar to the evaluation of comorbidities and laboratory data [46]. By integrating social support assessments into routine evaluations, practitioners may identify patients at higher risk of adverse outcomes due to insufficient support. This approach may guide interventions aimed at improving social support, such as connecting patients with community resources and involving family members in the care plan, which may enhance recovery and long-term outcomes for AMI patients [56].

### Limitations

This study has several limitations. Firstly, this was a single-center study in which data were collected retrospectively in a routine manner and not for the intention of this research. Also, among all AMI patients of the study cohort, only 38.7% had documentation of support type, perhaps indicating a reporting bias. Some of the study groups were of small sample sizes. Potentially relevant data were missing, such as socio-economic status/patient employment status, living conditions, medication regimen and adherence, and further details regarding the nature and quality of the social support provided. Patient functional status was assessed using the NS and not based on assessment of instrumental activities of daily living (IADL), given that it was not available. Additionally, support status was determined based on documentation at the time of the AMI event and is subject to change after hospital discharge. Lastly, we only examined all-cause mortality and did not explore cause-specific (such as cardiovascular) mortality and other morbidity indices, such as hospital readmissions and post-AMI quality of life.

## 5. Conclusions

This study underscores the critical role of social support in long-term mortality among patients following AMI. Patients without familial support faced a significantly elevated risk of mortality, independent of traditional clinical risk factors. Patient evaluation of support resources is integral following an AMI event for continued patient care and secondary prevention.

## Figures and Tables

**Table 1:** Baseline characteristics of the study population by study group.

Parameter	Value	Support Group					*p*
		1	2	3	4	5	
		(Employed Partner)	(Unemployed Partner)	(No Partner, Family Support)	(Benefit-Dependent)	(Non-Kin Support)	
*n*		458	823	1060	154	157	
**Demographics**							
Age, years	Mean (SD)	57.18 (10.05)	67.47 (12.77)	69.63 (13.91)	71.64 (14.05)	81.13 (10.23)	<0.001
	<65	372 (81.2)	335 (40.7)	393 (37.1)	52 (33.8)	11 (7.0)	<0.001
	65–75	67 (14.6)	238 (28.9)	255 (24.1)	31 (20.1)	20 (12.7)	
	≥75	19 (4.1)	250 (30.4)	412 (38.9)	71 (46.1)	126 (80.3)	
Sex	Male	370 (80.8)	672 (81.7)	556 (52.5)	95 (61.7)	62 (39.5)	<0.001
Ethnicity	Arab/Other	79 (17.2)	247 (30.0)	222 (20.9)	16 (10.4)	8 (5.1)	<0.001
**Cardiac diseases**							
Supraventricular arrhythmias		42 (9.2)	157 (19.1)	219 (20.7)	26 (16.9)	53 (33.8)	<0.001
Congestive heart failure		52 (11.4)	156 (19.0)	252 (23.8)	24 (15.6)	53 (33.8)	<0.001
Pulmonary heart disease		23 (5.0)	82 (10.0)	159 (15.0)	19 (12.3)	26 (16.6)	<0.001
Chronic ischemic heart disease		415 (90.6)	692 (84.1)	817 (77.1)	112 (72.7)	83 (52.9)	<0.001
History of myocardial infarction		89 (19.4)	151 (18.3)	184 (17.4)	27 (17.5)	30 (19.1)	0.892
History of PCI		98 (21.4)	195 (23.7)	224 (21.1)	34 (22.1)	27 (17.2)	0.405
History of CABG		40 (8.7)	123 (14.9)	119 (11.2)	19 (12.3)	21 (13.4)	0.017
**Cardiovascular risk factors**							
Renal diseases		18 (3.9)	88 (10.7)	129 (12.2)	14 (9.1)	29 (18.5)	<0.001
Diabetes mellitus		179 (39.1)	451 (54.8)	535 (50.5)	81 (52.6)	84 (53.5)	<0.001
Dyslipidemia		405 (88.4)	678 (82.4)	855 (80.7)	104 (67.5)	97 (61.8)	<0.001
Hypertension		228 (49.8)	498 (60.5)	640 (60.4)	92 (59.7)	91 (58.0)	0.002
Obesity		111 (24.2)	183 (22.2)	203 (19.2)	28 (18.2)	14 (8.9)	<0.001
Smoking		284 (62.0)	378 (45.9)	382 (36.0)	51 (33.1)	24 (15.3)	<0.001
Peripheral vascular disease		36 (7.9)	109 (13.2)	127 (12.0)	13 (8.4)	28 (17.8)	0.003
**Other disorders**							
COPD		21 (4.6)	104 (12.6)	125 (11.8)	16 (10.4)	22 (14.0)	<0.001
Malignancy		16 (3.5)	52 (6.3)	49 (4.6)	10 (6.5)	6 (3.8)	0.154
Anemia		124 (27.1)	335 (40.7)	491 (46.3)	76 (49.4)	98 (62.4)	<0.001
Neurological disorders		43 (9.4)	161 (19.6)	210 (19.8)	29 (18.8)	67 (42.7)	<0.001
Schizophrenia/psychosis		1 (0.2)	11 (1.3)	24 (2.3)	9 (5.8)	8 (5.1)	<0.001
Alcohol/drug addiction		6 (1.3)	12 (1.5)	30 (2.8)	11 (7.1)	1 (0.6)	<0.001
Dementia/Parkinson’s disease		4 (0.9)	46 (5.6)	86 (8.1)	14 (9.1)	57 (36.3)	<0.001
**Functional status**							
Physical condition	Good	326 (71.2)	425 (51.6)	451 (42.5)	54 (35.1)	18 (11.5)	<0.001
	Fair	124 (27.1)	339 (41.2)	504 (47.5)	77 (50.0)	85 (54.1)	
	Poor	8 (1.7)	52 (6.3)	91 (8.6)	23 (14.9)	45 (28.7)	
	Very bad	0	6 (0.7)	14 (1.3)	0	9 (5.7)	
Mental condition	Alert	454 (99.1)	796 (96.7)	1018 (96.0)	149 (96.8)	130 (82.8)	<0.001
	Apathetic	2 (0.4)	11 (1.3)	18 (1.7)	2 (1.3)	11 (7.0)	
	Confused	1 (0.2)	7 (0.9)	18 (1.7)	3 (1.9)	15 (9.6)	
	Stuporous	1 (0.2)	8 (1.0)	6 (0.6)	0	1 (0.6)	
Activity	Ambulant	323 (70.5)	384 (46.7)	392 (37)	49 (31.8)	7 (4.5)	<0.001
	Walks with help	122 (26.6)	380 (46.2)	590 (55.7)	98 (63.6)	98 (62.4)	
	Chairbound	10 (2.2)	32 (3.9)	44 (4.2)	3 (1.9)	26 (16.6)	
	Bedfast	3 (0.7)	26 (3.2)	34 (3.2)	4 (2.6)	26 (16.6)	
Mobility	Full	408 (89.1)	552 (67.1)	600 (56.6)	77 (50.0)	17 (10.8)	<0.001
	Slightly impaired	36 (7.9)	176 (21.4)	290 (27.4)	52 (33.8)	42 (26.8)	
	Very limited	11 (2.4)	65 (7.9)	135 (12.7)	19 (12.3)	69 (43.9)	
	Immobile	3 (0.7)	29 (3.5)	35 (3.3)	6 (3.9)	29 (18.5)	
Incontinence	None	437 (95.4)	703 (85.4)	851 (80.3)	119 (77.3)	59 (37.6)	<0.001
	Occasional	7 (1.5)	45 (5.5)	69 (6.5)	9 (5.8)	11 (7.0)	
	Usually urinary	10 (2.2)	40 (4.9)	79 (7.5)	11 (7.1)	33 (21.0)	
	Urinary and fecal	4 (0.9)	34 (4.1)	61 (5.8)	15 (9.7)	54 (34.4)	
Norton Scale	Mean (SD)	19.12 (1.57)	17.99 (2.52)	17.50 (2.67)	17.20 (2.60)	13.76 (3.27)	<0.001
	<16	22 (4.8)	144 (17.5)	269 (25.4)	40 (26.0)	114 (73.2)	<0.001
**Characteristics of AMI**							
Admitted/transposed to ICCU		377 (82.3)	535 (65.0)	603 (56.9)	73 (47.4)	32 (20.4)	<0.001
Type of AMI	STEMI	206 (45.0)	238 (28.9)	268 (25.3)	45 (29.2)	14 (8.9)	<0.001
Results of echocardiography *							
Severe LV dysfunction		51 (12.5)	108 (16.2)	122 (15.1)	14 (12.8)	16 (23.2)	0.152
LV hypertrophy		19 (4.7)	53 (7.9)	64 (7.9)	9 (8.3)	7 (10.1)	0.200
Mitral regurgitation		13 (3.2)	31 (4.6)	48 (5.9)	12 (11.0)	9 (13.0)	0.001
Measure of CAD **	No/non-significant	23 (5.7)	41 (6.7)	55 (8.0)	10 (11.0)	2 (6.7)	0.003
	One vessel	138 (34.2)	157 (25.7)	161 (23.4)	22 (24.2)	7 (23.3)	
	Two vessels	118 (29.2)	158 (25.8)	194 (28.2)	26 (28.6)	4 (13.3)	
	Three vessels/LM	125 (30.9)	256 (41.8)	278 (40.4)	33 (36.3)	17 (56.7)	
**Type of treatment**	Noninvasive	47 (10.3)	197 (23.9)	358 (33.8)	62 (40.3)	127 (80.9)	<0.001
	PCI	366 (79.9)	541 (65.7)	612 (57.7)	86 (55.8)	30 (19.1)	
	CABG	45 (9.8)	85 (10.3)	90 (8.5)	6 (3.9)	0	
**In-hospital course**							
Cardiac arrest		1 (0.2)	3 (0.4)	2 (0.2)	0	2 (1.3)	0.194
Cardiogenic shock		4 (0.9)	14 (1.7)	13 (1.2)	1 (0.6)	3 (1.9)	0.619
Intra-aortic balloon pulsation		6 (1.3)	17 (2.1)	7 (0.7)	2 (1.3)	0	0.045
Any form of pacing		6 (1.3)	18 (2.2)	15 (1.4)	3 (1.9)	2 (1.3)	0.670
Mechanical ventilation		8 (1.7)	20 (2.4)	33 (3.1)	2 (1.3)	7 (4.5)	0.232
Gastrointestinal bleeding		7 (1.5)	14 (1.7)	24 (2.3)	5 (3.2)	5 (3.2)	0.490
Blood transfusion		23 (5.0)	79 (9.6)	99 (9.3)	17 (11.0)	26.(16.6)	<0.001
Sepsis		3 (0.7)	9 (1.1)	15 (1.4)	1 (0.6)	4 (2.5)	0.353

Data are presented as numbers (percentage), unless specified otherwise. AMI—acute myocardial infarction; CABG—coronary artery bypass graft; CAD—coronary artery disease; COPD—chronic obstructive pulmonary disease; ICCU—intensive cardiac care unit; LM—left main (coronary artery); LV—left ventricular; PCI—percutaneous coronary intervention; SD—standard deviation; STEMI—ST-elevation myocardial infarction. * for those who underwent echocardiogram (*n* = 2061); ** for those who underwent angiography (*n* = 1825).

**Table 2:** Mortality indices according to study group.

Parameter	Support Group					*p*
	1	2	3	4	5	
	(Employed Partner)	(Unemployed Partner)	(No Partner, Family Support)	(Benefit-Dependent)	(Non-Kin Support)	
*n*	458	823	1060	154	157	
Mortality rate, *n* (%)	104 (22.7)	461 (56.0)	654 (61.7)	103 (66.9)	149 (94.9)	<0.001
Cumulative mortality	0.233	0.575	0.633	0.679	0.949	<0.001

**Table 3:** Multivariable model of the association of support type with long-term all-cause mortality after acute myocardial infarction.

Parameter	Value	AdjHR	(95% CI)	*p*
Support group	1 (employed partner)	1 (ref.)		
	2 (unemployed partner)	1.508	(1.207–1.883)	<0.001
	3 (no partner, family support)	1.540	(1.238–1.917)	<0.001
	4 (benefit-dependent)	1.644	(1.238–2.184)	<0.001
	5 (non-kin support)	2.201	(1.667–2.906)	<0.001
Age, years	<65	1 (ref.)		
	65–75	1.855	(1.576–2.182)	<0.001
	≥75	2.448	(2.078–2.884)	<0.001
Physical condition	4 vs. <4	1.236	(1.087–1.406)	0.001
Mental condition	4 vs. <4	1.738	(1.389–2.173)	<0.001
Activity	4 vs. <4	1.371	(1.164–1.616)	<0.001
Mobility	4 vs. <4	1.409	(1.212–1.639)	<0.001
Incontinence	4 vs. <4	1.227	(1.067–1.411)	0.004
Supraventricular arrhythmias		1.35	(1.196–1.522)	<0.001
Congestive heart failure		1.197	(1.060–1.353)	0.004
History of myocardial infarction		1.178	(1.033–1.342)	0.014
Renal diseases		1.484	(1.278–1.723)	<0.001
Diabetes mellitus		1.125	(1.007–1.257)	0.038
Hypertension		0.854	(0.764–0.955)	0.006
Obesity		0.85	(0.737–0.980)	0.025
Peripheral vascular disease		1.455	(1.263–1.677)	<0.001
COPD		1.48	(1.279–1.713)	<0.001
Neurological disorders		1.357	(1.203–1.530)	<0.001
Malignancy		1.86	(1.523–2.271)	<0.001
Anemia		1.267	(1.130–1.420)	<0.001
Alcohol/drug addiction		1.876	(1.322–2.664)	<0.001
Type of AMI	NSTEMI vs. STEMI	1.230	(1.058–1.431)	0.007
Severe LV dysfunction		1.351	(1.151–1.584)	<0.001
LV hypertrophy		1.244	(1.010–1.534)	0.040
Mitral regurgitation		1.536	(1.236–1.908)	<0.001
Type of treatment	Noninvasive	1 (ref.)		
	PCI	0.704	(0.617–0.803)	<0.001
	CABG	0.584	(0.455–0.749)	<0.001

AdjHR—adjusted hazard ratio; AMI—acute myocardial infarction; CABG—coronary artery bypass graft; CI—confidence interval; COPD—Chronic obstructive pulmonary disease; LV—left ventricular; NSTEMI—Non-ST-elevation myocardial infarction; PCI—percutaneous coronary intervention; ref.—reference group; STEMI—ST-elevation myocardial infarction.

## Data Availability

The data underlying this article will be shared upon reasonable request to the corresponding author.

## Supplementary Materials

The following supporting information can be downloaded at: https://www.mdpi.com/article/10.3390/jcdd12040147/s1, Figure S1: Study flow chart; Table S1: Baseline characteristics of the study population; Table S2: Distribution of support type by subgroup; Table S3: Association of support type with long-term all-cause mortality after acute myocardial infarction—subgroup multivariable analysis.
