# Supplementary material for: Social Support and 10-Year Mortality Following Acute Myocardial Infarction
Source: J Cardiovasc Dev Dis. 2025 Apr 10;12(4):147. doi: 10.3390/jcdd12040147 (PMC12028073; doi:10.3390/jcdd12040147)
Supplement: Supplementary file 1 [file jcdd-12-00147-s001.zip › jcdd-3466366-supplementary.pdf]

Supplementary Materials

Supplementary Figure S1. Study flow chart

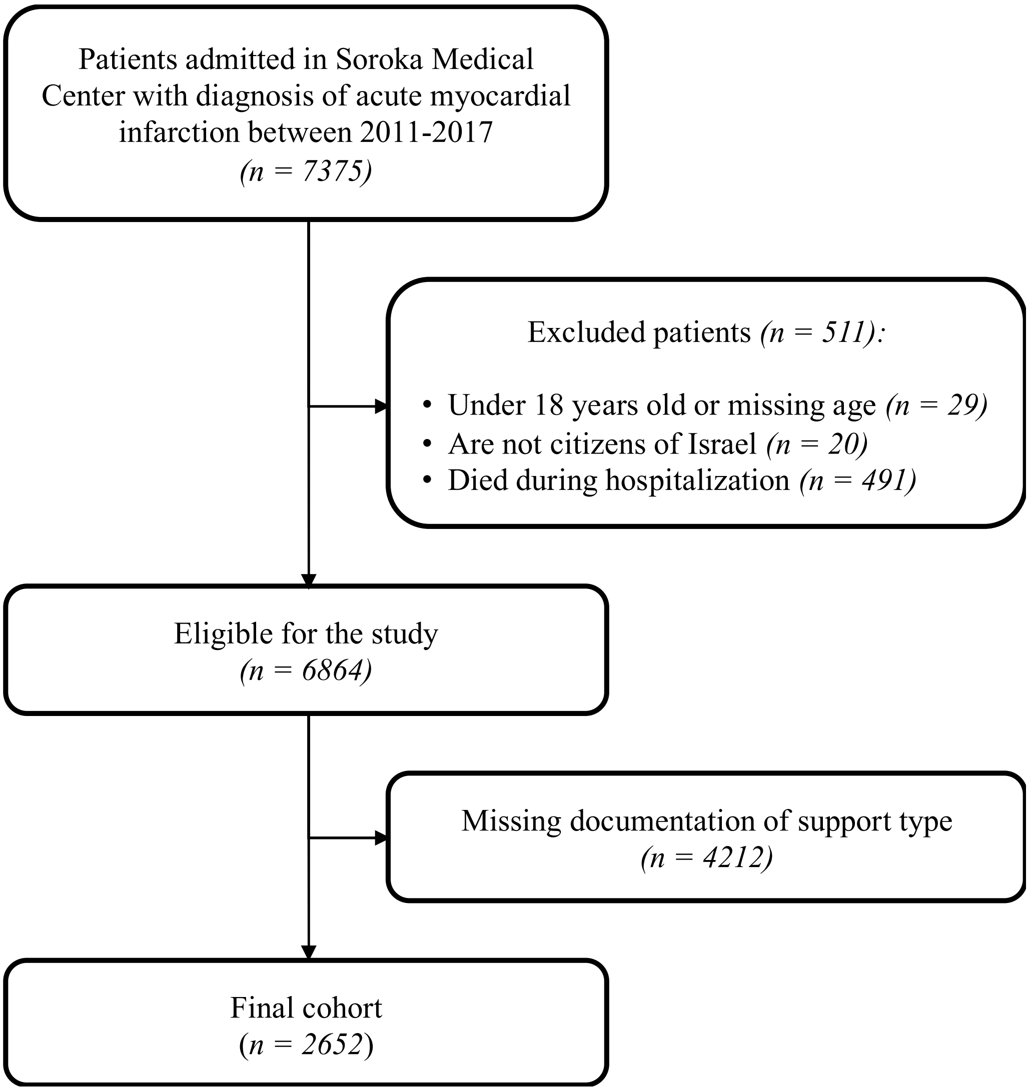

Supplementary Table S1. Baseline characteristics of the study population

Parameter	Value	Distribution* (n = 2652)
**Demographics**		
Age, Years	Mean (SD)	67.61 (13.97)
	<65	1163 (43.9)
	65-75	611 (23.0)
	≥75	878 (33.1)
Sex	Male	1755 (66.2)
Ethnicity	Arab / Other	572 (21.6)
**Cardiac diseases**		
Supraventricular arrhythmias		497 (18.7)
Congestive heart failure		537 (20.2)
Pulmonary heart disease		309 (11.7)
Chronic ischemic heart disease		2119 (79.9)
History of myocardial infarction		481 (18.1)
History of PCI		578 (21.8)
History of CABG		322 (12.1)
**Cardiovascular risk factors**		
Renal diseases		278 (10.5)
Diabetes mellitus		1330 (50.2)
Dyslipidemia		2139 (80.7)
Hypertension		1549 (58.4)
Obesity		539 (20.3)
Smoking		1119 (42.2)
Peripheral vascular disease		313 (11.8)
**Other disorders**		
COPD		288 (10.9)
Malignancy		133 (5.0)
Anemia		1124 (42.4)
Neurological disorders		510 (19.2)
Schizophrenia / psychosis		53 (2.0)
Alcohol / drug addiction		60 (2.3)
Dementia / Parkinson's disease		207 (7.8)
**Functional status**		
Physical condition	Good	1274 (48.0)
	Fair	1129 (42.6)
	Poor	219 (8.3)
	Very bad	29 (1.1)
Mental condition	Alert	2547 (96.0)
	Apathetic	44 (1.7)
	Confused	44 (1.7)
	Stuporous	16 (0.6)
Activity	Ambulant	1155 (43.6)
	Walks with help	1288 (48.6)
	Chairbound	115 (4.3)
	Bedfast	93 (3.5)
Mobility	Full	1654 (62.4)

	Slightly impaired	596 (22.5)
	Very limited	299 (11.3)
	Immobile	102 (3.8)
Incontinence	None	2169 (81.8)
	Occasional	141 (5.3)
	Usually urinary	173 (6.5)
	Urinary and Fecal	168 (6.3)
Norton Scale	Mean (SD)	17.69 (2.75)
	<16	589 (22.2)
**Characteristics of AMI**		
Admitted / transposed to ICCU		1620 (61.1)
Type of AMI	STEMI	771 (29.1)
**Results of echocardiography****		
Severe LV dysfunction		311/2061 (15.1)
LV hypertrophy		152/2061 (7.4)
Mitral regurgitation		113/2061 (5.5)
**Measure of CAD*****	No / non-significant	131/1825 (7.2)
	One vessel	485/1825 (26.6)
	Two vessels	500/1825 (27.4)
	Three vessels / LM	709/1825 (38.8)
**Type of treatment**	Noninvasive	791 (29.8)
	PCI	1635 (61.7)
	CABG	226 (8.5)
**In-hospital course**		
Cardiac arrest		8 (0.3)
Cardiogenic shock		35 (1.3)
Intra-aortic balloon pulsation		32 (1.2)
Any form of pacing		44 (1.7)
Mechanical ventilation		70 (2.6)
Gastrointestinal bleeding		55 (2.1)
Blood transfusion		244 (9.2)
Sepsis		32 (1.2)

* Data are presented as numbers (percentage), unless specified otherwise; **for those who underwent echocardiogram (n = 2061); ***for those who underwent angiography (n = 1825). AMI - acute myocardial infarction; AV - atrioventricular (block); CABG - coronary artery bypass graft; CAD - coronary arteries disease; COPD - Chronic obstructive pulmonary disease; ICCU – intensive cardiac care unit; LM - left main (coronary artery); LV - left ventricular; PCI - percutaneous coronary intervention; SD – standard deviation; STEMI – ST-elevation myocardial infarction.

Supplementary Table S2. Distribution of support type by subgroup

**Age**				
**Support Group** **n**	**Age <75 years** **1774**	**Age≥75 years** **878**	**Total** **2652**	**p**
1 (employed partner)	439 (24.7)	19 (2.2)	458 (17.3)	<0.001
2 (unemployed partner)	573 (32.3)	250 (28.5)	823 (31.0)	
3 (no partner, family support)	648 (36.5)	412 (46.9)	1060 (40.0)	
4 (benefit-dependent)	83 (4.7)	71 (8.1)	154 (5.8)	
5 (non-kin support)	31 (1.7)	126 (14.4)	157 (5.9)	
**Sex**				
**Support Group** **n**	**Women** **897**	**Men** **1755**	**Total** **2652**	**p**
1 (employed partner)	88 (9.8)	370 (21.1)	458 (17.3)	<0.001
2 (unemployed partner)	151 (16.8)	672 (38.3)	823 (31)	
3 (no partner, family support)	504 (56.2)	556 (31.7)	1060 (40.0)	
4 (benefit-dependent)	59 (6.6)	95 (5.4)	154 (5.8)	
5 (non-kin support)	95 (10.6)	62 (3.5)	157 (5.9)	
**Nationality**				
**Support Group** **n**	**Jews** **2080**	**Arabs** **572**	**Total** **2652**	**p**
1 (employed partner)	379 (18.2)	79 (13.8)	458 (17.3)	<0.001
2 (unemployed partner)	576 (27.7)	247 (43.2)	823 (31.0)	
3 (no partner, family support)	838 (40.3)	222 (38.8)	1060 (40.0)	
4 (benefit-dependent)	138 (6.6)	16 (2.8)	154 (5.8)	
5 (non-kin support)	149 (7.2)	8 (1.4)	157 (5.9)	
**Functional status**				
**Support Group** **n**	**Norton Scale ≥16** **2061**	**Norton Scale <16** **590**	**Total** **2651**	**p**
1 (employed partner)	436 (21.2)	22 (3.7)	458 (17.3)	<0.001
2 (unemployed partner)	678 (32.9)	144 (24.4)	822 (31.0)	
3 (no partner, family support)	791 (38.4)	269 (45.6)	1060 (40.0)	
4 (benefit-dependent)	114 (5.5)	40 (6.8)	154 (5.8)	
5 (non-kin support)	42 (2.0)	115 (19.5)	157 (5.9)	
**Age and sex**				
**Support Group**	**Age <75 years**	**Age ≥75 years**	**Total**	**p**
**Women: n**	**436**	**461**	**897**	
1 (employed partner)	84 (19.3)	4 (0.9)	88 (9.8)	<0.001

2 (unemployed partner)	89 (20.4)	62 (13.4)	151 (16.8)	
3 (no partner, family support)	232 (53.2)	272 (59)	504 (56.2)	
4 (benefit-dependent)	23 (5.3)	36 (7.8)	59 (6.6)	
5 (non-kin support)	8 (1.8)	87 (18.9)	95 (10.6)	
**Men: n**	**1338**	**417**	**1755**	
1 (employed partner)	355 (26.5)	15 (3.6)	370 (21.1)	
2 (unemployed partner)	484 (36.2)	188 (45.1)	672 (38.3)	
3 (no partner, family support)	416 (31.1)	140 (33.6)	556 (31.7)	<0.001
4 (benefit-dependent)	60 (4.5)	35 (8.4)	95 (5.4)	
5 (non-kin support)	23 (1.7)	39 (9.4)	62 (3.5)	
**Nationality and sex**				
**Support Group**	**Jews**	**Arabs**	**Total**	**p**
**Women: n**	**742**	**155**	**897**	
1 (employed partner)	67 (9.0)	21 (13.5)	88 (9.8)	
2 (unemployed partner)	124 (16.7)	27 (17.4)	151 (16.8)	
3 (no partner, family support)	401 (54.0)	103 (66.5)	504 (56.2)	<0.001
4 (benefit-dependent)	58 (7.8)	1 (0.6)	59 (6.6)	
5 (non-kin support)	92 (12.4)	3 (1.9)	95 (10.6)	
**Men: n**	**1338**	**417**	**1755**	
1 (employed partner)	312 (23.3)	58 (13.9)	370 (21.1)	
2 (unemployed partner)	452 (33.8)	220 (52.8)	672 (38.3)	
3 (no partner, family support)	437 (32.7)	119 (28.5)	556 (31.7)	<0.001
4 (benefit-dependent)	80 (6.0)	15 (3.6)	95 (5.4)	
5 (non-kin support)	57 (4.3)	5 (1.2)	62 (3.5)	

Data are presented as numbers (percentage).

Supplementary Table S3. Association of support type with long-term all-cause mortality after acute myocardial infarction – subgroup multivariable analysis

Age

Support Group	Age <75 years			Age ≥75 years		
	**AdjHR***	**(95% CI)**	**p**	**AdjHR***	**(95% CI)**	**p**
1 (employed partner)	1 (ref.)			1 (ref.)		
2 (unemployed partner)	1.367	(1.061 - 1.759)	0.015	1.697	(0.969 - 2.974)	0.064
3 (no partner, family support)	1.410	(1.103 - 1.803)	0.006	1.561	(0.896 - 2.720)	0.116
4 (benefit-dependent)	1.669	(1.148 - 2.427)	0.007	1.512	(0.827 - 2.766)	0.180
5 (non-kin support)	2.556	(1.577 - 4.143)	<0.001	1.989	(1.114 - 3.552)	0.020

Sex

Support Group	Women			Men		
	**AdjHR***	**(95% CI)**	**p**	**AdjHR***	**(95% CI)**	**p**
1 (employed partner)	1 (ref.)			1 (ref.)		
2 (unemployed partner)	1.406	(0.874 - 2.262)	0.160	1.496	(1.160 - 1.93)	0.002
3 (no partner, family support)	1.510	(0.963 - 2.366)	0.073	1.539	(1.188 - 1.992)	0.001
4 (benefit-dependent)	1.835	(1.086 - 3.101)	0.023	1.521	(1.061 - 2.181)	0.023
5 (non-kin support)	2.257	(1.358 - 3.751)	0.002	1.947	(1.334 - 2.843)	<0.001

Nationality

Support Group	Jews			Arabs		
	**AdjHR***	**(95% CI)**	**p**	**AdjHR***	**(95% CI)**	**p**
1 (employed partner)	1 (ref.)			1 (ref.)		
2 (unemployed partner)	1.502	(1.173 - 1.923)	0.001	1.679	(0.949 - 2.971)	0.075
3 (no partner, family support)	1.501	(1.180 - 1.908)	<0.001	1.825	(1.019 - 3.271)	0.043
4 (benefit-dependent)	1.552	(1.144 - 2.105)	0.005	3.702	(1.502 - 9.125)	0.004
5 (non-kin support)	2.136	(1.586 - 2.877)	<0.001	2.820	(0.961 - 8.278)	0.059

Functional status

Support Group	Norton Scale ≥16			Norton Scale <16		
	**AdjHR***	**(95% CI)**	**p**	**AdjHR***	**(95% CI)**	**p**
1 (employed partner)	1 (ref.)			1 (ref.)		
2 (unemployed partner)	1.454	(1.131 - 1.868)	0.003	1.067	(0.628 - 1.814)	0.811
3 (no partner, family support)	1.666	(1.305 - 2.128)	<0.001	1.040	(0.621 - 1.742)	0.882
4 (benefit-dependent)	1.754	(1.254 - 2.454)	0.001	1.177	(0.650 - 2.133)	0.590
5 (non-kin support)	2.106	(1.382 - 3.208)	<0.001	1.631	(0.952 - 2.796)	0.075

Age and Sex

Support Group	Age <75 years			Age ≥75 years		
	**AdjHR***	**(95% CI)**	**p**	**AdjHR***	**(95% CI)**	**p**
**Women:**						
1 (employed partner)	1 (ref.)			1 (ref.)		
2 (unemployed partner)	1.088	(0.609 - 1.944)	0.776	1.112	(0.371 - 3.330)	0.850

Support Group	AdjHR*	(95% CI)	p	AdjHR*	(95% CI)	p
3 (no partner, family support)	1.380	(0.820 - 2.320)	0.225	1.014	(0.348 - 2.950)	0.980
4 (benefit-dependent)	1.380	(0.682 - 2.793)	0.370	1.259	(0.405 - 3.918)	0.691
5 (non-kin support)	5.294	(2.051 - 13.665)	<0.001	1.323	(0.448 - 3.902)	0.613
**Men:**						
1 (employed partner)	1 (ref.)			1 (ref.)		
2 (unemployed partner)	1.449	(1.091 - 1.924)	0.010	1.355	(0.694 - 2.646)	0.374
3 (no partner, family support)	1.444	(1.082 - 1.928)	0.013	1.452	(0.739 - 2.855)	0.279
4 (benefit-dependent)	1.966	(1.237 - 3.124)	0.004	1.025	(0.483 - 2.177)	0.949
5 (non-kin support)	1.953	(1.083 - 3.523)	0.026	1.871	(0.891 - 3.927)	0.098

Nationality and Sex

Support Group	Jews			Arabs		
	**AdjHR***	**(95% CI)**	**p**	**AdjHR***	**(95% CI)**	**p**
**Women:**						
1 (employed partner)	1 (ref.)			1 (ref.)		
2 (unemployed partner)	1.236	(0.731 - 2.089)	0.429	2.144	(0.636 - 7.225)	0.219
3 (no partner, family support)	1.332	(0.809 - 2.193)	0.26	2.917	(0.952 - 8.932)	0.061
4 (benefit-dependent)	1.665	(0.949 - 2.923)	0.076	8.351	(0.742 - 93.993)	0.086
5 (non-kin support)	2.162	(1.239 - 3.770)	0.007	7.624	(1.280 - 45.392)	0.026
**Men:**						
1 (employed partner)	1 (ref.)			1 (ref.)		
2 (unemployed partner)	1.447	(1.087 - 1.925)	0.011	1.784	(0.897 - 3.548)	0.099
3 (no partner, family support)	1.564	(1.178 - 2.075)	0.002	2.003	(0.986 - 4.072)	0.055
4 (benefit-dependent)	1.513	(1.026 - 2.231)	0.036	4.716	(1.742 - 12.764)	0.002
5 (non-kin support)	2.628	(1.751 - 3.946)	<0.001	2.514	(0.505 - 12.52)	0.260

* Adjusted for the investigated baseline characteristics (see Table 3). AdjHR - adjusted hazard ratio- CI – confidence interval- ref. – reference group.
